# Syphilitic Consumption

**Published:** 1879-05

**Authors:** 


SYPHLITIC CONSUMPTION.—Prof. Schnitzter, of Vienna, in the *Wiener Med. Presse* has called attention to the much greater frequency of this disease than has been hitherto supposed. The symptoms strictly follow those of ordinary tuberculosis, and the diagnosis is often far from easy. The existence of syphlitic symptoms in any other organ or part in a consumptive should at once lead to the suspicion that the phthisis is of like origin; so also, should the confession of previous venereal infection. The recognition of the character of the disease is all important, as specific treatment in the earlier stages will generally effect a prompt cure. *Med. and Surg. Reporter.*

